# Data Resource Profile: Whole-Blood DNA Methylation Resource in Generation Scotland (MeGS)

**DOI:** 10.1093/ije/dyaf091

**Published:** 2025-07-09

**Authors:** Rosie M Walker, Daniel L McCartney, Kevin Carr, Michael Barber, Xueyi Shen, Archie Campbell, Elena Bernabeu, Emma Aitken, Angie Fawkes, Nicola Wrobel, Lee Murphy, Heather C Whalley, David M Howard, Mark J Adams, Konrad Rawlik, Pau Navarro, Albert Tenesa, Cathie L Sudlow, David J Porteous, Riccardo E Marioni, Andrew M McIntosh, Kathryn L Evans

**Affiliations:** Centre for Genomic and Experimental Medicine, Institute of Genetics and Cancer, University of Edinburgh, Edinburgh, United Kingdom; School of Psychology, University of Exeter, Exeter, United Kingdom; Centre for Genomic and Experimental Medicine, Institute of Genetics and Cancer, University of Edinburgh, Edinburgh, United Kingdom; Centre for Genomic and Experimental Medicine, Institute of Genetics and Cancer, University of Edinburgh, Edinburgh, United Kingdom; Medical Research Council Human Genetics Unit, Institute of Genetics and Cancer, University of Edinburgh, Edinburgh, United Kingdom; Division of Psychiatry, University of Edinburgh, Royal Edinburgh Hospital, Edinburgh, United Kingdom; Centre for Genomic and Experimental Medicine, Institute of Genetics and Cancer, University of Edinburgh, Edinburgh, United Kingdom; Usher Institute of Population Health Sciences and Informatics, University of Edinburgh, Edinburgh, United Kingdom; Centre for Genomic and Experimental Medicine, Institute of Genetics and Cancer, University of Edinburgh, Edinburgh, United Kingdom; Edinburgh Clinical Research Facility, University of Edinburgh, Western General Hospital, Edinburgh, United Kingdom; Edinburgh Clinical Research Facility, University of Edinburgh, Western General Hospital, Edinburgh, United Kingdom; Edinburgh Clinical Research Facility, University of Edinburgh, Western General Hospital, Edinburgh, United Kingdom; Edinburgh Clinical Research Facility, University of Edinburgh, Western General Hospital, Edinburgh, United Kingdom; Centre for Genomic and Experimental Medicine, Institute of Genetics and Cancer, University of Edinburgh, Edinburgh, United Kingdom; Division of Psychiatry, University of Edinburgh, Royal Edinburgh Hospital, Edinburgh, United Kingdom; Institute of Psychiatry, Psychology and Neuroscience, King's College London, United Kingdom; Division of Psychiatry, University of Edinburgh, Royal Edinburgh Hospital, Edinburgh, United Kingdom; Baillie Gifford Pandemic Science Hub, University of Edinburgh, Edinburgh, United Kingdom; Medical Research Council Human Genetics Unit, Institute of Genetics and Cancer, University of Edinburgh, Edinburgh, United Kingdom; The Roslin Institute, Royal (Dick) School of Veterinary Studies, University of Edinburgh, United Kingdom; The Roslin Institute, Royal (Dick) School of Veterinary Studies, University of Edinburgh, United Kingdom; Usher Institute of Population Health Sciences and Informatics, University of Edinburgh, Edinburgh, United Kingdom; Health Data Research UK, London, United Kingdom; Centre for Genomic and Experimental Medicine, Institute of Genetics and Cancer, University of Edinburgh, Edinburgh, United Kingdom; Centre for Genomic and Experimental Medicine, Institute of Genetics and Cancer, University of Edinburgh, Edinburgh, United Kingdom; Division of Psychiatry, University of Edinburgh, Royal Edinburgh Hospital, Edinburgh, United Kingdom; Centre for Genomic and Experimental Medicine, Institute of Genetics and Cancer, University of Edinburgh, Edinburgh, United Kingdom

**Keywords:** DNA methylation, Generation Scotland, epigenomics

Key Features

- We have generated whole-blood DNA methylation profiles from 18 869 Generation Scotland: Scottish Family Health Study (GS) participants, resulting in, at the time of writing, the largest single-cohort DNA methylation resource for basic biological and medical research: Methylation in Generation Scotland (MeGS).
- GS is a community- and family-based cohort, which recruited >24 000 participants from Scotland between 2006 and 2011. Comprehensive phenotype information, including detailed data on cognitive function, personality traits, and mental health, is available for all participants. The majority of GS participants (83%) have genome-wide single-nucleotide polymorphism genotype data (Illumina HumanOmniExpressExome-8 array v1.0 and v1.2).
- Over 97% of GS participants have given consent for health record linkage and re-contact.
- At baseline, blood-based DNA methylation was characterized at ∼850 000 sites across four waves by using the Illumina EPICv1 array. MeGS participants were aged between 17 and 99 years at the time of enrolment in GS.
- Blood-based DNA methylation EPICv1 array profiles collected at a follow-up appointment that took place 4.3–12.2 years (mean = 7.1 years) after baseline are also available for 796 MeGS participants.
- Access to MeGS for researchers and collaborators is via application to the GS Access Committee (access@generationscotland.org).

## Data resource basics

Methylation in Generation Scotland (MeGS), which was initiated in 2016, was established to allow the integration of whole-blood DNA methylation data with the rich phenotypic, genetic, and electronic health record linkage already available for Generation Scotland (GS): a population- and family-based cohort (*N* = 24 096 from 6862 families) [1–3]. DNA methylation is an epigenetic modification influenced by both genetic and environmental factors, making it an attractive candidate for investigating mechanisms underlying complex traits and disease. In some circumstances, DNA methylation is associated with the expression of nearby or, less often, distal genes. There is clear evidence for associations between variation in DNA methylation and health-related behaviours, complex phenotypes, and disease outcomes [4–11]. MeGS, which is located in Edinburgh, UK was primarily funded through Wellcome Trust support.

GS was established through a multi-institutional collaboration, involving Scottish medical schools and the National Health Service (NHS) [1, 2]. It is an ideal cohort for the development of clinical and research biomarkers for use in disease prevention, detection, and monitoring, as whole blood is available from almost all participants, whilst linkage to health and prescription records allows the assessment of associations with both past and future health outcomes. Moreover, as participants have provided permission for re-contact, the potential exists to add further longitudinal data points to MeGS, permitting studies of changes in methylation across the life course.

MeGS comprises blood-based DNA methylation profiles from 18 869 individuals, making it, at the time of writing, the largest published single-cohort methylation resource in the world. For 796 of these participants, a second blood DNA methylation profile (the longitudinal MeGS sample) is available from an appointment that took place between 2015 and 2018 as part of a sub-study called ‘Stratifying Resilience and Depression Longitudinally’ (STRADL). Key demographic information for MeGS is displayed in Table 1. The MeGS cohort, which has an average age of 47.12 years (SD 14.90 years), is broadly representative of the larger GS cohort, containing a higher proportion of females (58.8%), higher education levels, and lower deprivation levels than the general Scottish population (Table 1). However, the participants for whom DNA methylation from a second time point is available are older, have a greater average body mass index, and are from less-deprived localities than the baseline population (Table 1).

**Table 1.** Key demographic information for GS baseline and STRADL follow-up DNA methylation datasets.

	**GS baseline (*N* = 18** **869)**	STRADL follow-up (*N* = 796)
**Age (years)**		
Mean (SD)	47.12 (14.90)	56.49 (9.63)
Age range	17–99	20–82
**Sex [*n* (%)]**		
Males	7771 (41.1)	342 (43.0)
Females	11094 (58.8)	454 (57.0)
Not reported	0 (0)	3 (0.4)
**Education [years (%)]**		
0	7 (0.04)	0 (0)
1–4	50 (0.26)	4 (0.5)
5–9	546 (2.9)	16 (2.0)
10–11	4954 (26.25)	246 (30.9)
12–13	3888 (20.6)	171 (21.5)
14–15	2563 (13.58)	96 (12.1)
16–17	3525 (18.68)	137 (17.2)
18–19	1699 (9.0)	70 (8.8)
20–21	431 (2.28)	15 (1.89)
22–23	128 (0.68)	8 (1.0)
24+	62 (0.33)	2 (0.25)
Not reported/missing	1016 (5.4)	31 (3.89)
**Deprivation (SIMD rank)**		
Mean (SD)	3898 (1848.55)	4270 (1758.75)
**BMI (kg/m^2^)**		
Mean (SD)	26.67 (5.18)	28.07 (5.58)
Range	10.49–71.35	16.4–62.4
**Smoking [*n* (%)]**		
Ever	8630 (45.7)	326 (40.8)
Never	9636 (51.1)	402 (50.3)
Missing	603 (3.2)	72 (9)
**Ethnicity** ^a^ **[*n* (%)]**		
White	∼18 140 (∼96.1)	∼780 (∼97.9)
Mixed/other	<10 (∼0.05)	∼15 (∼1.9)
Missing	∼720 (∼3.8)	<10 (∼1.2)

SIMD provides a rank of small areas across Scotland (data zones) from most deprived (Rank 1) to least deprived (Rank 6976).

BMI, body mass index; SIMD, Scottish Index of Multiple Deprivation.

a For disclosure control purposes, counts for ethnicity have been approximated.

Recruitment to GS is ongoing (‘NextGenScot’, https://www.ed.ac.uk/generation-scotland) [12] with the aim of doubling the cohort size by recruiting additional members of existing families and new families, and lowering the minimum age of participation to 12 years. Questionnaires are administered via an online portal and funding is in place to profile salivary DNA methylation in >10 000 of the newly recruited participants via the Illumina Methylation Screening Array (MSA). In parallel with recruiting new participants, members of the original GS study (including individuals in MeGS) have been encouraged to register on the portal. This will permit long-term longitudinal active data collection of MeGS individuals, in addition to passive data collection mediated by linkage to health records.

## Data collected

### Baseline data and sampling

The data and biological samples collected at the baseline clinic visit have been described in detail previously [2]. Participants completed a comprehensive preclinical questionnaire capturing information on various demographic variables, social characteristics, personal behaviours, cognitive and mental health, and self-reported health data. Participants also attended a clinic, at which physical and cognitive measurements were acquired, along with blood and urine samples.

The full range of phenotypic, clinical, and biochemical data available for GS (and, therefore, MeGS) participants has been described previously [1–3] and is searchable via the GS data dictionary (https://datashare.ed.ac.uk/handle/10283/2988). Genome-wide genotype information is available for the majority of MeGS participants (99.9%) [13]. When recruited between 2006 and 2011, GS participants provided blood or saliva samples (for biochemistry and cryopreservation) and a urine sample, meaning that it is possible to measure additional biomarkers in this cohort. As a condition of access by external researchers, any resource generated from GS must be returned to the study to be made available to the wider research community. This has resulted in the availability of cardiac and inflammatory biomarker data (NT-proBNP, GDF-15, cardiac troponins, and C-reactive protein) [14]. Furthermore, additional layers of omics data, including mass spectrometry proteomics data (*N* = 15 818; *N*_MeGS_ = 14 671) and Nightingale metabolomics data (*N* = 2907; *N*_MeGS_ = 2745), have also been made available to GS through this route.

A subset of the MeGS cohort (44.4%) is also enrolled in the STRADL sub-study, in which questionnaires examining psychological resilience, coping style, threatening life experiences, and physical and mental health were administered to 21 525 eligible GS participants, with 9618 respondents (8379 in MeGS) [15]. Of the 8379 participants, 1033 attended an in-person appointment at which additional clinical and cognitive assessments were performed; blood, saliva, and hair samples were collected; and neuroimaging was performed [16]. A second DNA methylation measure was obtained for 796 individuals who both were in the baseline DNA methylation dataset and attended this STRADL clinic appointment.

Plasma levels of 4058 proteins were measured by using SOMAscan^®^ V.4 technology in 839 MeGS participants from samples collected at the STRADL appointment [17]. Hormone levels were assayed from hair samples acquired as part of STRADL in 1009 individuals, of whom 732 have methylation at both baseline and longitudinal time points. A subset of MeGS participants (*N* = 4233) also took part in the CovidLife surveys, which examined the effects of COVID-19 measures on health and wellbeing [18].

### Data linkage

Ninety-seven percent of GS participants consented to linkage to their NHS Scotland records. Linked datasets include hospital admissions from the Scottish Morbidity Record (SMR), dispensed prescription information, MIDAS dental data, and the Scottish Drug Misuse Database. SMR linkage includes general hospital admissions, maternity and neonatal data, psychiatric admissions, and diabetes and cancer registries. Mortality data are also available through linkage to the National Records of Scotland. New linkages, such as Scottish Medical Imaging, radiology reports, and retinal scans, are planned to continue following participants over time [3]. Linkage to primary care is also available for GS, albeit currently limited to a subset of 6486 MeGS participants due to the constraints of data controller permission requirements.

### Sample processing

DNA methylation was profiled by using the Infinium MethylationEPIC BeadChip v1 (Illumina Inc., CA), which measures methylation at >850 000 sites across the human genome, covering 99% of RefSeq genes and providing enhanced coverage of regulatory regions compared with previous Illumina methylation arrays. Peripheral blood samples were collected in Ethylenediaminetetraacetic acid (EDTA) tubes according to standard procedures and DNA extracted by using Nucleon BACC3 extraction kits. Whole-blood genomic DNA (500 ng) was treated with sodium bisulphite by using the EZ-96 DNA Methylation Kit (Zymo Research, Irvine, California), following the manufacturer’s instructions. DNA methylation was then measured by using the MethylationEPIC BeadChip, according to the manufacturer’s instructions. Array scanning was performed by using a HiScan scanner (Illumina Inc., San Diego, CA) and an initial review of the data quality was carried out by using GenomeStudio (version 2011.1).

DNA methylation was profiled in four waves between 2016 and 2021 (Table 2). For all waves, the raw intensity data (IDAT) files were read into R, by using functions within minfi v.1.20.2–1.42.0 [19].

**Table 2.** Details of samples processed in each of the four methylation waves.

	Wave 1	Wave 2		Wave 3		Wave 4	Combined
**Date prepared**	Q4 2016–Q1 2017	Q1 2017–Q2 2017		Q4 2018–Q1 2019		Q3 2021	Q2 2023
**Study time point**	Baseline	Baseline	Longitudinal	Baseline	Longitudinal	Baseline	Baseline

**Number of samples (pre-QC)**	5200	465	520	4597	373	9082	20 232
**Number of samples (post-QC)**	5087	459	501	4450	295	8873	18 869
**Number of probes (post-QC)**	860 925	859 730		856 671		854 642	851 610

Q1 = Quarter 1 (January–March).

Q2 = Quarter 2 (April–June).

Q3 = Quarter 3 (July–September).

Q4 = Quarter 4 (October–December).

QC: quality control.

Quality control (QC) and normalization were applied to each wave separately, at the time it was produced, to enable insights from these novel data as they emerged. Prior to commencing QC of the first wave, 10 samples were removed, as they were derived from saliva and were mistakenly submitted for whole-blood methylation profiling. Three further samples were removed due to inaccurate self-report data (i.e. answering ‘Yes’ to all self-reported conditions). Another sample was removed, as information from a separate study highlighted that they were likely to be XXY. For the first wave, QC was performed by using shinyMethyl v1.10.0 [20]. First, technical outliers were removed based on visual inspection of a plot of the log median intensity of the methylated versus unmethylated signal for each array. ShinyMethyl’s control probe plots were then inspected to identify outliers. Next, samples for which the sex predicted from the methylation data (based on the difference between the median copy number intensity for the Y chromosome and the median copy number intensity for the X chromosome) did not match the participant’s self-reported sex were removed. Multidimensional scaling (MDS) plots were inspected for any additional sample outliers but none was detected. The pfilter function within wateRmelon v.1.18.0 [21] was used to remove: (i) samples in which >1% of the cytosine–phosphate–guanine (CpG) sites had a detection *P*-value of >0.05; (ii) probes with a beadcount of <3 in >5% of the samples; and (iii) probes for which >0.5% of the samples had a detection *P*-value of >0.05. Proportions of six white blood cell types (monocytes, granulocytes, CD 4+ T cells, CD 8+ T cells, B cells, and natural killer cells) were estimated by using minfi’s implementation of the Houseman algorithm [22] with Reinius *et al.*’s peripheral blood reference data [23].

The QC of the DNA methylation data produced in Waves 2–4 was carried out by using both meffil vs.1.1.0 and 1.1.2 [24] and shinyMethyl vs.1.14.0 and 1.30.0 [20]. Meffil was used to perform dye-bias and background correction by using the ‘noob’ method [25] and exclude: samples affected by a strong dye bias or issues affecting bisulphite conversion (using default thresholds); samples for which the median methylated signal intensity was >3 SD lower than expected; and samples in which the methylation-predicted sex deviated from the self-reported sex. Deviations between methylation-predicted sex and self-reported sex were also assessed by using shinyMethyl’s sex-prediction function. ShinyMethyl was additionally used to plot the output of all control probes to permit the detection of outliers by visual inspection. Following these sample removal steps, meffil was used to filter poorly performing samples and probes. Samples were removed if they had >0.5% CpG sites with a detection *P*-value of >0.01. Once the poorly performing samples had been removed, meffil was rerun on the remaining dataset to identify poorly performing probes. These were defined as probes with a beadcount of <3 in >5% of the samples and/or >1% of the samples had a detection *P*-value of >0.01. White blood cell proportions were estimated by using meffil’s implementation of the Houseman algorithm [22] with Reinius *et al.*’s peripheral blood reference data [23].

**Figure 1.**
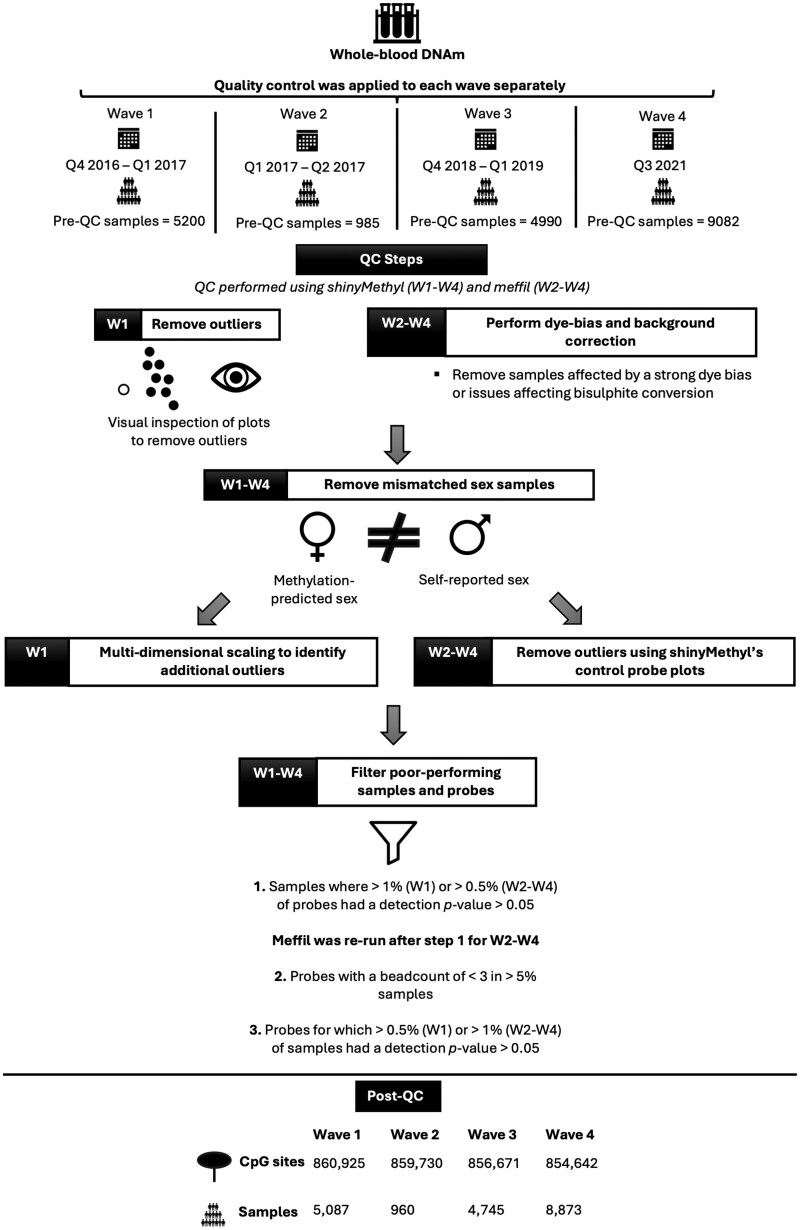
Schematic diagram outlining the QC process for DNA methylation data in GS. DNA methylation was profiled from whole-blood samples in four waves. QC was performed for each wave separately. The QC of Wave 1 was performed exclusively by using shinyMethyl whilst the QC of Waves 2–4 was performed by using both meffil and shinyMethyl. The figure indicates when the DNA methylation profiling took place (Q = quarter) and how many samples were present before QC. For all waves, steps were implemented to remove poorly performing samples and probes, and to remove samples in which the sex predicted from the methylation data differed from the self-reported sex. Waves 2–4 were subject to dye-bias and background correction as part of the ‘noob’ normalization process implemented in meffil, whilst, for Wave 1, background correction was performed during the subsequent ‘dasen’ normalization step. The final number of CpG sites and samples are outlined for each wave.

Following QC, the data from Y-chromosome probes were removed and the data from each of the four waves were normalized separately by using the dasen method from the R package wateRmelon v.2.2.0 [21]. Dasen involves adjusting the background difference between Type I assays (which interrogate methylated and unmethylated CpGs with separate probes) and Type II assays (which interrogate methylated and unmethylated CpGs with a single probe), by adding the offset between Type I and Type II probe intensities to Type I intensities. Between-array quantile normalization is then performed for the methylated and unmethylated signal intensities separately, with Type I and Type II assays being normalized separately. The dasen-normalized beta-values were logit-transformed to methylation M-values by using the beta2M function in the R package lumi v.2.30.0 [26]. Following normalization, X-chromosome probes were excluded from the dataset together with probes that had been predicted to bind suboptimally according to Zhou *et al.* [27] or McCartney *et al.* [28]. The normalized data (with and without the data from the X-chromosome probes) were inspected by using MDS plots to identify any remaining outlier samples. MDS plots were generated from subsets of the (i) 10 000 and (ii) 100 000 most variable probes, colour-coding by methylation-processing batch (whereby samples in the same batch underwent bisulfite conversion, array hybridization, and scanning at the same time) and sex. Visual inspection of these plots identified 40 male outliers in Wave 3 who did not cluster with the other males. As a precaution, these samples were removed. For Waves 2 and 3, the normalized datasets were subsequently separated into baseline and longitudinal sample sets. A jointly normalized dataset is also available, whereby the four separately QC’d waves were combined and normalized by using dasen. The dasen-normalized beta-values were converted into M-values as described above. A summary of the QC and normalization steps is provided in Fig. 1.

## Data resource use

The analysis of DNA methylation data can provide novel insights into the mechanisms underlying diseases, health traits, and basic biological phenomena; identify biomarkers; and improve the prediction of future health outcomes. MeGS has contributed to research efforts across all these domains, resulting in 63 publications to date (Supplementary Table S1). Several methylome-wide association studies (MWASs) have been carried out by using MeGS to identify associations between DNA methylation and a range of complex traits and diseases (Supplementary Table S1). As an illustrative example, we report here MWASs for two personality traits: neuroticism and extraversion, which have not been assessed previously in a large sample. MWASs were performed by using *limma* v.3.54 [29]. Methylation levels in M-values (pre-corrected for relatedness by using a genomic relationship matrix) at 752 721 CpG sites were included as the dependent variable and the independent variable was the score for a personality trait, as measured by using the Eysenck Personality Questionnaire (neuroticism: *n *= 18 788, extraversion: *n *= 18 783). The following model was fitted for each trait:

*DNA methylation (corrected M-values) ∼ personality trait score + age + sex + methylation-processing batch + estimated blood cell counts + methylation-derived smoking score + 20 methylation-based principal components*.

**Figure 2.**
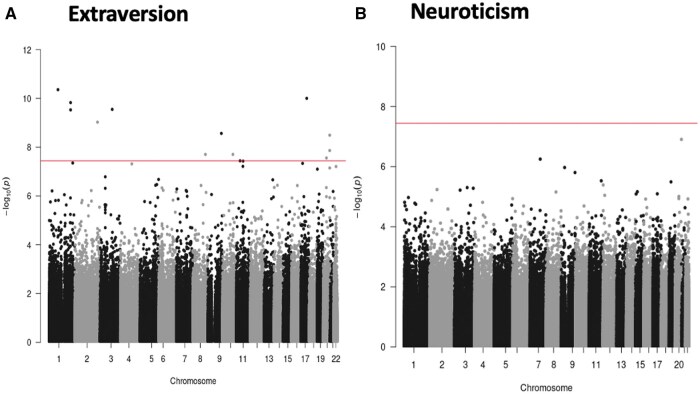
Manhattan plots of MWASs of (A) extraversion and (B) neuroticism. Each point represents a CpG site tested for association. –log_10_  *P*-values [–log_10_(p)] are presented along the *y*-axis with chromosome and genomic position along the *x*-axis. The horizontal line represents the epigenome-wide significant threshold of *P* = 3.6 × 10^−8^.

A threshold of *P *< 3.6 × 10^−8^ was used to identify methylome-wide significant associations [30]. Fourteen CpG sites were identified as significantly associated with extraversion (Fig. 2A and Table 3). No sites were significantly associated with neuroticism (Fig. 2B). Sensitivity analyses were performed without covarying for the smoking score, identifying 70 genome-wide significant loci for neuroticism and 19 loci for extraversion. Results of these analyses are provided in Supplementary Tables S2 and S3.

**Table 3.** Differentially methylated sites associated with extraversion at *P* < 3.6× 10^−8^.

Probe ID	Effect	SE	*P*	Gene
cg02947519	–9.14 × 10^–3^	1.41 × 10^–3^	1.05 × 10^–10^	–
cg10501210	6.20 × 10^–3^	9.73 × 10^–4^	1.96 × 10^–10^	–
cg15393490	8.93 × 10^–3^	1.47 × 10^–3^	1.18 × 10^–9^	–
cg05083539	8.24 × 10^–3^	1.36 × 10^–3^	1.46 × 10^–9^	*PKND*; *TMBIM1*
cg00329615	7.99 × 10^–3^	1.30 × 10^–3^	7.86 × 10^–10^	*IGSF11*
cg06690548	–1.45 × 10^–2^	1.96 × 10^–3^	1.82 × 10^–13^	*SLC7A11*
cg10585061	8.29 × 10^–3^	1.50 × 10^–3^	3.29 × 10^–8^	–
cg01182455	6.92 × 10^–3^	1.20 × 10^–3^	8.67 × 10^–9^	–
cg11376147	–7.51 × 10^–3^	1.06 × 10^–3^	1.20 × 10^–12^	*SLC43A1*
cg10861637	6.85 × 10^–3^	1.12 × 10^–3^	8.79 × 10^–10^	*CLTC*
cg11851174	8.67 × 10^–3^	1.52 × 10^–3^	1.34 × 10^–8^	*RAI1*
cg02231404	–8.97 × 10^–3^	1.50 × 10^–3^	2.45 × 10^–9^	*SOX18*
cg04881720	–9.88 × 10^–3^	1.67 × 10^–3^	3.66 × 10^–9^	*SOX18*
cg22138735	–8.49 × 10^–3^	1.52 × 10^–3^	2.60 × 10^–8^	*SOX18*

MeGS has also been utilized in the development of DNA methylation predictors of health outcomes [4], complex traits [9], and blood protein levels [17]. The resource has also contributed to large-scale methylation quantitative trait loci (meQTL) consortia efforts [31], as well as multiple methodological papers. Epigenetic clocks have also been calculated from MeGS and have been made available to researchers as derived phenotypes. A comprehensive overview of the utilization of the MeGS resource is provided in the Supplementary Data.

## Strengths and weaknesses

### Strengths

MeGS is derived from the richly phenotyped GS cohort and was, at the time of writing, the largest single-cohort-based DNA methylation data resource. The use of the Illumina EPICv1 array, which almost doubles the coverage of its predecessor, the 450K array, is a further advantage over similar datasets [32]. Individuals in GS have been extensively phenotyped and the cohort is particularly well suited for studies on mental health and cognitive phenotypes. In addition, record linkage to routine health datasets further increases the scope for investigations of both prevalent and incident diseases and traits. Finally, the ability to re-contact participants for future studies permits longitudinal assessment. The within-sample relatedness of the dataset can be utilized to investigate, e.g., parent-of-origin effects on DNA methylation [33]. When analysing complex traits, however, relatedness may impact statistical power. Accounting for relatedness via mixed-effects modelling or limiting analyses to an unrelated subset mitigates this issue. The decision to analyse the four waves jointly or separately is likely to vary according to the specific research questions being addressed. Factors such as the benefits of analysing a large discovery sample versus splitting the data into test and replication samples should be considered on a trait-by-trait basis. Moreover, for some phenotypes, including diseases that occur with low frequency, it may be necessary to analyse the waves jointly in order to achieve adequate statistical power.

### Weaknesses

The EPICv1 array characterizes only ∼4% of the methylome and cannot distinguish between DNA methylation and hydroxymethylation; however, it covers 99% of RefSeq genes and has increased coverage of regulatory elements, such as enhancers, compared with previous Illumina methylation arrays. The EPICv1 platform has now also been superseded by the EPICv2 and lower-coverage MSA. Although there is substantial overlap between the MSA, EPICv1, and EPICv2 arrays, there may be downstream implications on the utility of MeGS for meta-analyses and replication studies across platforms. While blood is arguably not always the most mechanistically relevant tissue, the relative ease of obtaining peripheral blood samples makes it an appropriate tissue for developing biomarkers for prediction, monitoring disease course, and measuring the impact of treatment. In common with many other volunteer-based studies, GS has relatively small numbers of participants of non-white ancestry and more deprived backgrounds, and is not fully representative of the broader (Scottish) population. The comparatively small size of the STRADL follow-up subsample limits the utility of the resource for longitudinal DNAm analyses. Phenotypic bias may be present in this subset due to a higher lifetime history of mood disorders. However, it has been noted that, at the time of recruitment, these participants were in good psychological health [16]. Additionally, this sample has a geographical bias, with clinic recruitment focusing on participants residing proximal to sites in Dundee and Aberdeen. However, there is the potential to generate an enhanced longitudinal resource in the future, given that the cohort has consent for re-contact from the majority of baseline participants.

## Data resource access

Researchers wishing to access the MeGS resource and wider GS study data can do so by submitting an access application form to access@generationscotland.org (contact person Prof Riccardo E. Marioni). The full range of phenotypic, clinical, and biochemical data available for GS (and, therefore, MeGS) participants is searchable via the GS data dictionary (https://datashare.ed.ac.uk/handle/10283/2988). Access applications are subject to review through GS access processes, which ensure that all research using the resource aims to benefit the health and wellbeing of patients and the public. Approved projects are subject to a Data & Materials Transfer Agreement (DMTA) or commercial contract. Full information on the access procedure including application forms and DMTA templates is available at https://www.ed.ac.uk/generation-scotland/for-researchers/access. Data dictionaries describing the full GS resource are available online at https://datashare.ed.ac.uk/handle/10283/2988.

## Ethics approval

GS obtained ethical approval from the NHS Tayside Committee on Medical Research Ethics on behalf of the NHS (reference: 05/S1401/89) and has Research Tissue Bank Status (reference: 20/ES/0021). All components of STRADL received formal, national ethical approval from the NHS Tayside committee on research ethics (reference 14/SS/0039).

## Supplementary Material

dyaf091_Supplementary_Data

## Data Availability

Please see ‘Data resource access’ above.

## Supplementary data

Conflict of interest: D.L.M. is an employee of Optima Partners Ltd. L.M. has received speaker and consultancy fees from Illumina. A.M.M. has received research support from Eli Lilly, Janssen, and the Sackler Foundation. A.M.M. has also received speaker fees from Illumina and Janssen and consulting fees. R.E.M. has received speaker fees from Illumina. C.L.S. is Director of the British Heart Foundation Data Science Centre and Chief Scientist of the multifunder institute, Health Data Research UK.
