# Supplementary material for: Data Resource Profile: Whole-Blood DNA Methylation Resource in Generation Scotland (MeGS)
Source: Int J Epidemiol. 2025 Jul 9;54(4):dyaf091. doi: 10.1093/ije/dyaf091 (PMC12240559; doi:10.1093/ije/dyaf091)
Supplement: dyaf091_Supplementary_Data [file dyaf091_supplementary_data.zip › ije-2024-05-0702-File005.docx]

**Supplementary Data**

**Data Resource Use: Extended Information**

- **Prediction of health outcomes and complex traits:** Barbu et al. (2022) [1] showed that a risk score calculated from methylation data explained 1.75% of the variance in major depressive disorder (MDD). McCartney et al. (2018) [2] derived DNA methylation predictors for 10 modifiable health and lifestyle factors and showed that a DNA methylation predictor of body mass index (BMI), when used in conjunction with a polygenic risk score, could explain approximately twice as much trait variance compared to the polygenic risk score alone. Cheng et al (2023) [3] have used MeGS to augment 10-year risk prediction of diabetes. Previous work on subsets of the MeGS baseline data has included the identification of methylation sites associated with: genetic risk factors for depression [4]; antidepressant treatment [5]; alcohol use disorder [6]; cognitive ability [7]; and risk factors for dementia [8–10]. MeGS has also contributed to collaborative meta-analyses to investigate DNA methylation associations with ageing [11], aggression [12] and chronic kidney disease [13]. In addition, the longitudinal dataset (i.e. methylation data acquired as part of STRADL) was used to generate signatures for 17 protein markers of brain health [14]. Finally, Chybowska et al. demonstrated the utility of methylation-derived EpiScores and composite measures of these scores to identify CVD risk, independent of traditional risk factors [15].
- **Validation of proxies for blood-based protein levels:** Gadd et al. related DNA methylation-based predictors of 109 protein levels (EpiScores) to incident health outcomes over 14 years. Using a subset of the MeGS cohort, they identified 130 EpiScore-disease associations, highlighting the potential of DNA methylation-based scores for disease prediction and risk stratification [16].
- **Epigenetic clocks:** The difference between a person’s actual age and their age predicted from their methylation data (age acceleration) provides a measure of biological ageing, which has been shown to be predictive of multiple health outcomes and all-cause mortality [17,18]. Using MeGS, we have demonstrated significant associations between age acceleration and several health-related traits, including BMI, smoking, socioeconomic status, and brain health [19]. In addition, we have shown that local CpG density affects the trajectory and variance of age-associated DNA methylation changes [20]. We performed genome-wide association study meta-analyses of four epigenetic clocks and discovered evidence for shared genetic loci associated with the Horvath clock and expression of lipid metabolism and immune function genes [11]. Finally, using the MeGS cohort, Bernabeu et al. derived a DNA methylation-based predictor of chronological age with a median absolute error of 1.7 years, outperforming existing predictors by between 1.8 and 6.4 years [21]. Epigenetic clocks have been calculated for MeGS using the UCLA DNA methylation age calculator [22] and are available for researchers via the Generation Scotland data access process.
- **Improving understanding of basic biological mechanisms:** MeGS has contributed to a recent large-scale effort to map methylation quantitative trait loci (meQTL) [23]. This represents the most well-powered meQTL cataloguing effort to-date and resulted in the identification of > 270,000 independent meQTLs. The family-based nature of MeGS has facilitated studies into parent-of-origin effects on DNA methylation [24,25].
- **Methodological papers:** MeGS has been used to develop a Bayesian inference-based approach to analysis of methylation data [26], and has demonstrated the utility of Whatman FTA® cards for collecting and storing blood samples for DNA methylation profiling [27]. MeGS has also contributed to a study assessing variability in DNA methylation-based predictors of age and BMI, when integrating multiple DNA methylation datasets.[28]. This study highlighted the importance of selecting an appropriate normalisation method for combining datasets and generating epigenetic signatures [29].

**MWAS of Neuroticism and Extraversion: Sensitivity Analysis Results**

***Table S2*: Differentially Methylated CpGs associated with Extraversion at p < 3.6x10^-8^*, without covarying for a methylation-derived smoking score***

| **Probe ID** | **Effect** | **SE** | **P** | **Gene** |
| --- | --- | --- | --- | --- |
| cg10501210 | 0.0062 | 0.0010 | 8.43 X 10^-11^ | *-* |
| cg02947519 | -0.0090 | 0.0014 | 1.07 X 10^-10^ | *-* |
| cg15393490 | 0.0091 | 0.0014 | 2.54 X 10^-10^ | *-* |
| cg01557798 | -0.0066 | 0.0012 | 1.97 X 10^-8^ | *OBSCN* |
| cg05083539 | 0.0083 | 0.0013 | 6.66 X 10^-10^ | *PNKD;TMBIM1* |
| cg00329615 | 0.0077 | 0.0013 | 2.04 X 10^-9^ | *IGSF11* |
| cg06690548 | -0.0146 | 0.0019 | 5.41 X 10^-14^ | *SLC7A11* |
| cg20589883 | 0.0089 | 0.0016 | 2.76 X 10^-8^ | *ELOVL6* |
| cg10585061 | 0.0085 | 0.0015 | 8.28 X 10^-9^ | *-* |
| cg01182455 | 0.0069 | 0.0012 | 5.71 X 10^-9^ | *-* |
| cg07504977 | 0.0087 | 0.0015 | 4.51 X 10^-9^ | *-* |
| cg11376147 | -0.0076 | 0.0010 | 2.65 X 10^-13^ | *SLC43A1* |
| cg15208139 | 0.0077 | 0.0013 | 5.28 X 10^-9^ | *LDLRAD3* |
| cg19458497 | 0.0088 | 0.0016 | 2.66 X 10^-8^ | *ATL3* |
| cg10861637 | 0.0071 | 0.0011 | 1.33 X 10^-10^ | *CLTC* |
| cg04881720 | -0.0101 | 0.0017 | 8.79 X 10^-10^ | *SOX18* |
| cg02231404 | -0.0086 | 0.0015 | 5.68 X 10^-9^ | *SOX18* |
| cg15796392 | 0.0079 | 0.0014 | 1.07 X 10^-8^ | *NOL4L* |
| cg22138735 | -0.0083 | 0.0015 | 2.96 X 10^-8^ | *SOX18* |

***Table S3:* Differentially Methylated sites associated with Neuroticism at p < 3.6x10^-8^*, without covarying for a methylation-derived smoking score***

| **Probe ID** | **Effect** | **SE** | **P** | **Gene** |
| --- | --- | --- | --- | --- |
| cg09935388 | -0.0170 | 0.0021 | 9.33 X 10^-16^ | *GFI1* |
| cg27537125 | -0.0090 | 0.0012 | 2.68 X 10^-13^ | *-* |
| cg27215690 | -0.0135 | 0.0019 | 3.08 X 10^-12^ | *-* |
| cg04535902 | -0.0141 | 0.0021 | 4.95 X 10^-11^ | *GFI1* |
| cg27650500 | -0.0086 | 0.0014 | 2.20 X 10^-10^ | *-* |
| cg25189904 | -0.0135 | 0.0022 | 3.67 X 10^-10^ | *GNG12* |
| cg12876356 | -0.0137 | 0.0022 | 8.10 X 10^-10^ | *GFI1* |
| cg05603985 | -0.0091 | 0.0016 | 5.62 X 10^-9^ | *SKI* |
| cg10399789 | -0.0125 | 0.0022 | 7.80 X 10^-9^ | *GFI1* |
| cg18146737 | -0.0129 | 0.0023 | 1.26 X 10^-8^ | *GFI1* |
| cg09662411 | -0.0120 | 0.0022 | 2.61 X 10^-8^ | *GFI1* |
| cg04885881 | -0.0093 | 0.0017 | 3.29 X 10^-8^ | *-* |
| cg21566642 | -0.0195 | 0.0021 | 3.98 X 10^-20^ | *-* |
| cg01940273 | -0.0179 | 0.0021 | 1.78 X 10^-17^ | *-* |
| cg06644428 | -0.0169 | 0.0021 | 7.16 X 10^-16^ | *-* |
| cg03329539 | -0.0116 | 0.0017 | 1.51 X 10^-11^ | *-* |
| cg13193840 | -0.0124 | 0.0020 | 1.17 X 10^-9^ | *-* |
| cg23079012 | -0.0129 | 0.0022 | 3.76 X 10^-9^ | *-* |
| cg27241845 | -0.0108 | 0.0019 | 1.62 X 10^-8^ | *-* |
| cg07995927 | -0.0094 | 0.0017 | 2.47 X 10^-8^ | *TANK* |
| cg04414766 | 0.0130 | 0.0021 | 7.61 X 10^-10^ | *-* |
| cg23480021 | 0.0124 | 0.0021 | 3.47 X 10^-9^ | *-* |
| cg02920129 | -0.0122 | 0.0021 | 4.16 X 10^-9^ | *ZNF385D* |
| cg16615151 | -0.0101 | 0.0017 | 4.45 X 10^-9^ | *PLCXD2* |
| cg09945032 | -0.0107 | 0.0019 | 9.76 X 10^-9^ | *-* |
| cg05575921 | -0.0212 | 0.0021 | 2.64 X 10^-23^ | *AHRR* |
| cg21161138 | -0.0196 | 0.0021 | 8.47 X 10^-20^ | *AHRR* |
| cg25648203 | -0.0153 | 0.0021 | 3.98 X 10^-13^ | *AHRR* |
| cg26703534 | -0.0140 | 0.0020 | 9.00 X 10^-12^ | *AHRR* |
| cg14580211 | -0.0094 | 0.0017 | 2.21 X 10^-8^ | *C5orf62* |
| cg13039251 | 0.0104 | 0.0019 | 2.67 X 10^-8^ | *PDZD2* |
| cg14466441 | -0.0140 | 0.0021 | 2.98 X 10^-11^ | *-* |
| cg24859433 | -0.0118 | 0.0020 | 4.36 X 10^-9^ | *-* |
| cg16190265 | -0.0114 | 0.0018 | 1.18 X 10^-10^ | *PRKAR1B* |
| cg21322436 | -0.0142 | 0.0022 | 1.50 X 10^-10^ | *CNTNAP2* |
| cg25949550 | -0.0104 | 0.0017 | 3.48 X 10^-10^ | *CNTNAP2* |
| cg12803068 | 0.0125 | 0.0021 | 3.27 X 10^-9^ | *MYO1G* |
| cg09022230 | -0.0107 | 0.0018 | 4.29 X 10^-9^ | *TNRC18* |
| cg05009104 | 0.0117 | 0.0021 | 1.74 X 10^-8^ | *MYO1G* |
| cg24838345 | -0.0118 | 0.0021 | 2.42 X 10^-8^ | *MTSS1* |
| cg18497489 | 0.0092 | 0.0017 | 3.41 X 10^-8^ | *-* |
| cg03450842 | -0.0107 | 0.0019 | 2.64 X 10^-8^ | *ZMIZ1* |
| cg14391737 | -0.0154 | 0.0019 | 3.73 X 10^-16^ | *PRSS23* |
| cg19885130 | -0.0111 | 0.0017 | 1.11 X 10^-10^ | *LRP5* |
| cg09578155 | -0.0109 | 0.0017 | 4.68 X 10^-10^ | *LRP5* |
| cg21611682 | -0.0116 | 0.0019 | 2.25 X 10^-9^ | *LRP5* |
| cg11660018 | -0.0102 | 0.0018 | 1.94 X 10^-8^ | *PRSS23* |
| cg13784607 | -0.0086 | 0.0016 | 2.70 X 10^-8^ | *CELF1* |
| cg02583484 | -0.0112 | 0.0017 | 3.50 X 10^-11^ | *HNRNPA1;HNRPA1L-2* |
| cg09842685 | -0.0122 | 0.0022 | 2.31 X 10^-8^ | *-* |
| cg25845814 | -0.0125 | 0.0017 | 4.23 X 10^-14^ | *MIR4505;ELMSAN1* |
| cg25001882 | -0.0111 | 0.0016 | 2.45 X 10^-12^ | *-* |
| cg02738868 | -0.0071 | 0.0012 | 9.93 X 10^-10^ | *ELMSAN1* |
| cg04902004 | -0.0097 | 0.0018 | 3.17 X 10^-8^ | *GPR68* |
| cg18110140 | -0.0140 | 0.0020 | 1.54 X 10^-12^ | *-* |
| cg24947681 | -0.0094 | 0.0016 | 1.82 X 10^-9^ | - |
| cg17739917 | -0.0185 | 0.0017 | 2.58 X 10^-26^ | RARA |
| cg19572487 | -0.0113 | 0.0016 | 3.90 X 10^-13^ | RARA |
| cg24087280 | 0.0083 | 0.0013 | 1.52 X 10^-10^ | SAMD14 |
| cg00819417 | -0.0101 | 0.0018 | 1.88 X 10^-8^ | NF1 |
| cg22675726 | -0.0112 | 0.0019 | 2.78 X 10^-9^ | MYOM1 |
| cg03636183 | -0.0183 | 0.0021 | 1.86 X 10^-18^ | F2RL3 |
| cg21911711 | -0.0123 | 0.0018 | 1.21 X 10^-11^ | F2RL3 |
| cg15159987 | -0.0092 | 0.0016 | 8.77 X 10^-9^ | CPAMD8 |
| cg10765427 | -0.0112 | 0.0020 | 2.33 X 10^-8^ | CPAMD8 |
| cg24797066 | -0.0067 | 0.0010 | 4.98 X 10^-11^ | - |
| cg07339236 | -0.0126 | 0.0019 | 1.00 X 10^-10^ | ATP9A |
| cg17332125 | -0.0126 | 0.0022 | 1.12 X 10^-8^ | *-* |
| cg11643740 | 0.0111 | 0.0020 | 1.75 X 10^-8^ | *-* |
| cg05086879 | -0.0160 | 0.0021 | 8.31 X 10^-15^ | MGAT3 |

**References**

1. Barbu MC, Amador C, Kwong ASF, Shen X, Adams MJ, Howard DM, et al. Complex trait methylation scores in the prediction of major depressive disorder. EBioMedicine. 2022;79:104000.

2. McCartney DL, Hillary RF, Stevenson AJ, Ritchie SJ, Walker RM, Zhang Q, et al. Epigenetic prediction of complex traits and death. Genome Biology. 2018;19:136.

3. Cheng Y, Gadd DA, Gieger C, Monterrubio-Gómez K, Zhang Y, Berta I, et al. Development and validation of DNA methylation scores in two European cohorts augment 10-year risk prediction of type 2 diabetes. Nat Aging. 2023;3:450–8.

4. Barbu MC, Shen X, Walker RM, Howard DM, Evans KL, Whalley HC, et al. Epigenetic prediction of major depressive disorder. Mol Psychiatry. 2021;26:5112–23.

5. Barbu MC, Huider F, Campbell A, Amador C, Adams MJ, Lynall M-E, et al. Methylome-wide association study of antidepressant use in Generation Scotland and the Netherlands Twin Register implicates the innate immune system. Mol Psychiatry. 2022;27:1647–57.

6. Lohoff FW, Clarke T-K, Kaminsky ZA, Walker RM, Bermingham ML, Jung J, et al. Epigenome-wide association study of alcohol consumption in N = 8161 individuals and relevance to alcohol use disorder pathophysiology: identification of the cystine/glutamate transporter SLC7A11 as a top target. Mol Psychiatry. 2022;27:1754–64.

7. McCartney DL, Hillary RF, Conole ELS, Banos DT, Gadd DA, Walker RM, et al. Blood-based epigenome-wide analyses of cognitive abilities. Genome Biology. 2022;23:26.

8. Walker RM, Bermingham ML, Vaher K, Morris SW, Clarke T-K, Bretherick AD, et al. Epigenome-wide analyses identify DNA methylation signatures of dementia risk. Alzheimers Dement (Amst). 2020;12:e12078.

9. Walker RM, Vaher K, Bermingham ML, Morris SW, Bretherick AD, Zeng Y, et al. Identification of epigenome-wide DNA methylation differences between carriers of APOE ε4 and APOE ε2 alleles. Genome Med. 2021;13:1.

10. Mur J, McCartney DL, Walker RM, Campbell A, Bermingham ML, Morris SW, et al. DNA methylation in APOE: The relationship with Alzheimer’s and with cardiovascular health. Alzheimers Dement (N Y). 2020;6:e12026.

11. McCartney DL, Min JL, Richmond RC, Lu AT, Sobczyk MK, Davies G, et al. Genome-wide association studies identify 137 genetic loci for DNA methylation biomarkers of aging. Genome Biol. 2021;22:194.

12. van Dongen J, Hagenbeek FA, Suderman M, Roetman PJ, Sugden K, Chiocchetti AG, et al. DNA methylation signatures of aggression and closely related constructs: A meta-analysis of epigenome-wide studies across the lifespan. Mol Psychiatry. 2021;26:2148–62.

13. Schlosser P, Tin A, Matias-Garcia PR, Thio CHL, Joehanes R, Liu H, et al. Meta-analyses identify DNA methylation associated with kidney function and damage. Nat Commun. 2021;12:7174.

14. Gadd DA, Hillary RF, McCartney DL, Shi L, Stolicyn A, Robertson NA, et al. Integrated methylome and phenome study of the circulating proteome reveals markers pertinent to brain health. Nat Commun. 2022;13:4670.

15. Chybowska AD, Gadd DA, Cheng Y, Bernabeu E, Campbell A, Walker RM, et al. Epigenetic Contributions to Clinical Risk Prediction of Cardiovascular Disease. Circ Genom Precis Med. 2024;17:e004265.

16. Gadd DA, Hillary RF, McCartney DL, Zaghlool SB, Stevenson AJ, Cheng Y, et al. Epigenetic scores for the circulating proteome as tools for disease prediction. Lo YD, Ferrucci L, editors. eLife. 2022;11:e71802.

17. Horvath S, Raj K. DNA methylation-based biomarkers and the epigenetic clock theory of ageing. Nat Rev Genet. 2018;19:371–84.

18. DNA methylation age of blood predicts all-cause mortality in later life | Genome Biology | Full Text [Internet]. [cited 2024 Dec 16]. Available from: https://genomebiology.biomedcentral.com/articles/10.1186/s13059-015-0584-6

19. Hillary RF, Stevenson AJ, McCartney DL, Campbell A, Walker RM, Howard DM, et al. Epigenetic measures of ageing predict the prevalence and incidence of leading causes of death and disease burden. Clinical Epigenetics. 2020;12:115.

20. Higham J, Kerr L, Zhang Q, Walker RM, Harris SE, Howard DM, et al. Local CpG density affects the trajectory and variance of age-associated DNA methylation changes. Genome Biol. 2022;23:1–28.

21. Bernabeu E, McCartney DL, Gadd DA, Hillary RF, Lu AT, Murphy L, et al. Refining epigenetic prediction of chronological and biological age. Genome Medicine. 2023;15:12.

22. Horvath S. DNA methylation age of human tissues and cell types. Genome Biology. 2013;14:3156.

23. Min JL, Hemani G, Hannon E, Dekkers KF, Castillo-Fernandez J, Luijk R, et al. Genomic and phenotypic insights from an atlas of genetic effects on DNA methylation. Nat Genet. 2021;53:1311–21.

24. Zeng Y, Amador C, Xia C, Marioni R, Sproul D, Walker RM, et al. Parent of origin genetic effects on methylation in humans are common and influence complex trait variation. Nat Commun. 2019;10:1383.

25. Zeng Y, Amador C, Gao C, Walker RM, Morris SW, Campbell A, et al. Lifestyle and Genetic Factors Modify Parent-of-Origin Effects on the Human Methylome. EBioMedicine. 2021;74:103730.

26. Bayesian reassessment of the epigenetic architecture of complex traits | Nature Communications [Internet]. [cited 2024 Dec 16]. Available from: https://www.nature.com/articles/s41467-020-16520-1

27. Walker RM, MacGillivray L, McCafferty S, Wrobel N, Murphy L, Kerr SM, et al. Assessment of dried blood spots for DNA methylation profiling. Wellcome Open Res. 2019;4:44.

28. Merzbacher C, Ryan B, Goldsborough T, Hillary RF, Campbell A, Murphy L, et al. Integration of datasets for individual prediction of DNA methylation-based biomarkers. Genome Biol. 2023;24:278.

29. Pidsley R, Zotenko E, Peters TJ, Lawrence MG, Risbridger GP, Molloy P, et al. Critical evaluation of the Illumina MethylationEPIC BeadChip microarray for whole-genome DNA methylation profiling. Genome Biol. 2016;17:208.
